# Effects of phylogenetic reconstruction method on the robustness of species delimitation using single-locus data

**DOI:** 10.1111/2041-210X.12246

**Published:** 2014-10-29

**Authors:** Cuong Q Tang, Aelys M Humphreys, Diego Fontaneto, Timothy G Barraclough, Emmanuel Paradis

**Affiliations:** 1 Department of Life Sciences, Imperial College LondonAscot, Berkshire, SL5 7PY, UK; 2 Department of Ecology, Environment and Plant Sciences, Stockholm University10691, Stockholm, Sweden; 3 National Research Council, Institute of Ecosystem Study28922, Verbania Pallanza, Italy

**Keywords:** coalescent, DNA barcoding, GMYC, metabarcoding, molecular dating, NGS, OTU, PTP, speciation, species delimitation

## Abstract

1. Coalescent-based species delimitation methods combine population genetic and phylogenetic theory to provide an objective means for delineating evolutionarily significant units of diversity. The generalised mixed Yule coalescent (GMYC) and the Poisson tree process (PTP) are methods that use ultrametric (GMYC or PTP) or non-ultrametric (PTP) gene trees as input, intended for use mostly with single-locus data such as DNA barcodes.
2. Here, we assess how robust the GMYC and PTP are to different phylogenetic reconstruction and branch smoothing methods. We reconstruct over 400 ultrametric trees using up to 30 different combinations of phylogenetic and smoothing methods and perform over 2000 separate species delimitation analyses across 16 empirical data sets. We then assess how variable diversity estimates are, in terms of richness and identity, with respect to species delimitation, phylogenetic and smoothing methods.
3. The PTP method generally generates diversity estimates that are more robust to different phylogenetic methods. The GMYC is more sensitive, but provides consistent estimates for BEAST trees. The lower consistency of GMYC estimates is likely a result of differences among gene trees introduced by the smoothing step. Unresolved nodes (real anomalies or methodological artefacts) affect both GMYC and PTP estimates, but have a greater effect on GMYC estimates. Branch smoothing is a difficult step and perhaps an underappreciated source of bias that may be widespread among studies of diversity and diversification.
4. Nevertheless, careful choice of phylogenetic method does produce equivalent PTP and GMYC diversity estimates. We recommend simultaneous use of the PTP model with any model-based gene tree (e.g. RAxML) and GMYC approaches with BEAST trees for obtaining species hypotheses.

## Introduction

Species are a fundamental unit for many fields of biology, yet their identification and delimitation are rarely straightforward (Hebert. 2003). Molecular techniques allow for rapid and broad assessment of diversity of poorly known groups or where traditional techniques are difficult (Blaxter 2003; Tang. 2012; Fontaneto 2014). Well-established metrics for species delimitation exist (see Sites & Marshall 2003; Birky. 2005; Flot, Couloux & Tillier 2010; Puillandre. 2012a) but only a few are grounded in evolutionary theory and do not require *a priori* hypotheses regarding species entities (O'Meara 2010; Yang & Rannala 2010). Fewer still are designed for large-scale single-locus marker surveys (Fujisawa & Barraclough 2013; Zhang. 2013). With the increased frequency of DNA taxonomy studies and their potential marriage with next generation sequencing technologies (NGS – Creer. 2010), there is a need to determine potential sources of bias on diversity estimates. Here, we evaluate robustness of the generalised mixed Yule coalescent model (GMYC) and the Poisson tree process (PTP) species delimitation methods to different approaches of phylogenetic reconstruction of the gene trees. Robustness was assessed by how topological and branch length variation introduced by phylogenetic methods influences delimitation estimates in terms of species richness and identity.

A special branch of phylogenetic species delimitation (see Sites & Marshall 2003) is coalescent-based species delimitation methods (Pons. 2006; Fontaneto. 2007; Zhang. 2013), which combine coalescent theory with diversification models to infer the transition point between population and species-level processes on a gene tree. These approaches provide objective, clade-specific threshold(s) with which to delimit evolutionarily significant units (ESUs) of diversity (akin to species, as defined by the Evolutionary Species Concept – Simpson 1951). These methods provide an alternative to operational taxonomic unit (OTU) picking methods, which rely on arbitrary, clade-specific sequence similarity thresholds (Barraclough. 2009).

The GMYC is one of the most popular coalescent-based species delimitation methods and is designed for single-locus data (Fujisawa & Barraclough 2013; although it can be used with concatenated-loci data, Pons. 2006; Fontaneto. 2007) and has been used to describe new species (Birky. 2011). The method separately models the fit of Yule (pure birth; Yule 1925) and coalescent processes (Hudson 1990) to an ultrametric tree to define the transition from species-level to population-level processes, used to delimit ESUs. The PTP (Zhang. 2013) is a recently developed method that models speciation and coalescent events relative to numbers of substitutions rather than time, and uses heuristic algorithms to identify the most likely classification of branches into population and species-level processes, used to delimit ESUs. This approach assumes either that substitutions are clocklike or, if substitution rates vary across the tree, that coalescent and speciation events occur at a constant rate per substitution event, rather than per unit of time. The key advantage of the PTP, however, is that it is devised for non-ultrametric trees.

Several studies have evaluated factors that could bias accuracy of the GMYC and PTP. For the GMYC, simulation studies have addressed the effects of various aspects of sampling (Papadopoulou. 2008; Bergsten. 2012; Reid & Carstens 2012; Talavera, Dinca & Vila 2013), population size and speciation rates (Esselstyn. 2012; Fujisawa & Barraclough 2013). For the PTP, simulations have been used to evaluate the effect of birth rates (i.e. evolutionary distances between species) and sampling unevenness (Zhang. 2013). Less attention has been paid to the influence of different phylogenetic methods for reconstructing the underlying gene tree. For coalescent-based species delimitation, phylogenetic and branch smoothing (defined as methods that correct rate heterogeneity to make ultrametric, clocklike trees) methodology are potentially large sources of bias if branch length and topological variation is introduced by different phylogenetic methods, for example, by different treatment of unresolved nodes and rate heterogeneity. Zero-length branches introduce infinite (logarithmic) branching rate artefacts that might bias species delimitation and underestimate (early placement of the threshold) or overestimate (recent placement) species diversity (GMYC), and heterogeneity in the rate of molecular evolution among lineages would violate the assumption that branching events can be modelled against substitutions directly (PTP). It is well known that different methods of rate smoothing introduce variability in branch lengths (Drummond & Suchard 2010) that can ultimately affect inferences made from the tree (Rutschmann 2006); artificially variable branch lengths might therefore result in variable diversity estimates with the GMYC. A previous assessment of the effect of phylogenetic methods on GMYC ESU estimates showed that certain method combinations perform poorly (Talavera, Dinca & Vila 2013), but is not clear whether this is generally true.

The GMYC, in combination with at least 11 different phylogenetic and 9 smoothing methods (Table S1), has been used in over 150 studies. BEAST (Drummond & Rambaut 2007) is the most popular software for obtaining gene trees (48·9%), followed by MrBayes (25% – Ronquist. 2012) and RAxML (8·3% – Stamatakis 2006). BEAST is also the most popular software for rate smoothing (53·3%), followed by r8s (28·5% – Sanderson 2003), PATHd8 (6·7% – Britton. 2007) and *chronopl* (5·5% – Paradis, Claude & Strimmer 2004). It is not clear from the literature why one particular phylogenetic method is favoured. Is BEAST chosen (i) due to historical preference, (ii) because a posterior sample of trees is desired, (iii) because it does not require a *post hoc* rate-smoothing step, or (iv) because it provides more accurate species hypotheses than other methods? We address the latter issue for both the GMYC and PTP by systematically evaluating their performance given different phylogenetic methods across several data sets.

We evaluate the GMYC and PTP methods using cytochrome *c* oxidase subunit 1 (COI) data sets, first, where the species boundaries and diversity are relatively well known: cowries (Meyer & Paulay 2005), *Drosophila* spp. and Romanian butterflies (Dinca. 2011). Secondly, we compare the methods using 13 COI data sets of Rotifera, a phylum where the taxonomy is much less resolved, the sampling not as comprehensive, and where the benefit of DNA taxonomy is expected to be the greatest. We provide guidelines for maximising the robustness of species hypotheses based on single-locus data with respect to phylogenetic method.

## Materials and methods

### Data sets and gene trees

We compiled over 12 000 COI sequences forming 16 data sets (Table S2), corresponding mostly to genera (Rotifera + *Drosophila*) but also a family (Cypraeidae [cowries]; Meyer & Paulay 2005) and a comprehensive geographical sample comprising several families (99% of Romanian butterfly species; Dinca. 2011). Tree reconstruction followed standard protocols (Data S1; Fig. S1): (i) align sequences with out-groups (Table S3) using MAFFT v6.814b (Katoh, Asimenos & Toh 2009), (ii) remove non-unique haplotypes (for comparability the same matrix was used for all analyses, although this step is not necessary prior to generation of BEAST trees, see Talavera, Dinca & Vila 2013), (iii) reconstruct gene trees and (iv) make gene trees ultrametric. Gene trees were generated using distance (UPGMA – Sokal & Michener 1958; neighbour joining – Saitou & Nei 1987), maximum likelihood (GARLI – Zwickl 2006; RAxML – Stamatakis 2006; PhyML – Guindon. 2010) and Bayesian inference (MrBayes – Huelsenbeck & Ronquist 2001; BEAST – Drummond & Rambaut 2007). *Post hoc* branch smoothing (not necessary for BEAST and UPGMA trees) was performed using the R 2.15.2 (R Core Team 2012) package ape 3.0.7 functions (*chronopl* and *chronos* – Paradis, Claude & Strimmer 2004), PATHd8 (Britton. 2007) and r8s (Sanderson 2003).

### Unresolved nodes and rate heterogeneity

The presence of unresolved nodes and rate heterogeneity was measured directly from the trees. For each non-ultrametric gene tree, rate heterogeneity was measured as the standard deviation of the root to tip distances, where a greater standard deviation signifies greater rate heterogeneity. Analysis of BEAST trees was used to quantify whether the different species delimitation methods lead to different diversity estimates also where there are no unresolved nodes.

### Species delimitation

The GMYC method with a single threshold (ST-GMYC), multiple thresholds (MT-GMYC; Monaghan. 2009; Fujisawa & Barraclough 2013) and a multimodel approach (MM-GMYC; Powell 2012; Fujisawa & Barraclough 2013) was applied to each ultrametric gene tree using the splits 1·0–11 (Ezard, Fujisawa & Barraclough 2009) R package. PTP analyses were performed using its webserver (http://species.h-its.org/). For each clade, up to 25 different GMYC and 30 PTP estimates were made. Primarily, the PTP analysis was used with non-ultrametric gene trees (PTP-raw: trees without *post hoc* smoothing, as intended by Zhang. 2013), but smoothed trees were also used (PTP-all: all trees) for a direct comparison with the GMYC input trees.

### Performance variation among methods – Species richness

The deviance of each ESU estimate from the expected diversity was gauged using the absolute difference between observed (ESU_*X*_) and expected (ESU_expected_) diversity, standardised among data sets by dividing by the average diversity of the focal data set (ESU_meanA_: including the focal ESU estimate). ESU_expected_ was either obtained from the morphological species count (ESU_morph_) or the average of the species counts from across all trees (ESU_meanB_: excluding the focal ESU estimate). For the three data sets where the species boundaries have been better evaluated, the morphological species count was determined using either the GenBank species name (*Drosophila* and Romanian butterflies) or expert advice (cowries; C. Meyer pers. comm.). In the absence of a reliable taxonomic species count for the 13 Rotifera clades, ESU_meanB_ was used as a conservative estimate of species richness. The use of ESU_meanB_ as a proxy for ESU_expected_ was validated by the relationship between the residual variation derived from ESU_morph_ and from ESU_mean_ for the non-Rotifera data sets (File S1). Residual variation was determined for each gene tree and species delimitation method (see File S2 for examples of the calculations).

### Performance variation among methods – Species identity

Correspondence between ESUs and ESU_morph_, in terms of species identity, was evaluated for the three non-Rotifera data sets. For each species delimitation estimate, the number of morphospecies that were split, lumped, or an exact match to an ESU_morph_ was counted. Exact matches are where an ESU contains all species from a single morphospecies and no other. Morphospecies are split if they are found in more than one ESU and lumped if multiple morphospecies are present within a single ESU. These counts were performed for ST-GMYC, MT-GMYC, PTP-all and PTP-raw, but not for MM-GMYC because the method returns non-integers.

### Factors influencing residual variation of species richness and lumping and splitting of morphospecies

Generalised linear mixed models (GLMM; Bates. 2014) with a Poisson error structure were used to ascertain how residual variation varies with species delimitation methods, combination of phylogenetic and smoothing methods, rate heterogeneity, and presence of unresolved nodes. Three GLMMs were used to ask: For (i) all trees and (ii) BEAST trees, how does residual variation vary with species delimitation and phylogenetic methods? (iii) For trees with *post hoc* smoothing, how does residual variation vary with species delimitation and phylogenetic methods, presence of unresolved nodes and rate heterogeneity? For each of the models, residual variation was used as the response variable and clade was blocked out as a random effect.

A generalised linear model (GLM) with a quasibinomial error structure was used to assess if the proportion of morphospecies that are an exact match to an ESU (response variable) differed among species delimitation methods, clades and combination of phylogenetic and smoothing methods (explanatory variables).

For each of the models, significant differences among the levels were identified using *post hoc* Tukey HSD tests (multcomp 1.3-1 R package – Hothorn, Bretz & Westfall 2008). All analyses were performed in R.

## Results

For each of the 16 clades, ten gene trees (4x BEAST, MrBayes, GARLI, PhyML, RAxML, NJ and UPGMA) and 25 ultrametric trees were generated. MrBayes analysis of the cowrie data set (1459 tips) failed to converge, leading to a total of 159 gene trees and 396 ultrametric trees. These were analysed using the ST-GMYC (396 analyses), MT-GMYC (374 analyses) and MM-GMYC (286 analyses). For the cowries, only the ST-GMYC was performed owing to computational demands; MT-GMYC ran for over a week on a 3GHz processor with 8GB RAM without reaching a local likelihood optimum. The reduced number of analyses for the MM-GMYC is due to the method not accommodating trees with unresolved nodes without manual input (the logarithm of zero-length branches produces an infinite branching rate), which would not have been achievable within the scope of the present study. In total, 475 PTP and 1056 GMYC analyses were performed (Table S3).

### Species richness

**Figure 1:**
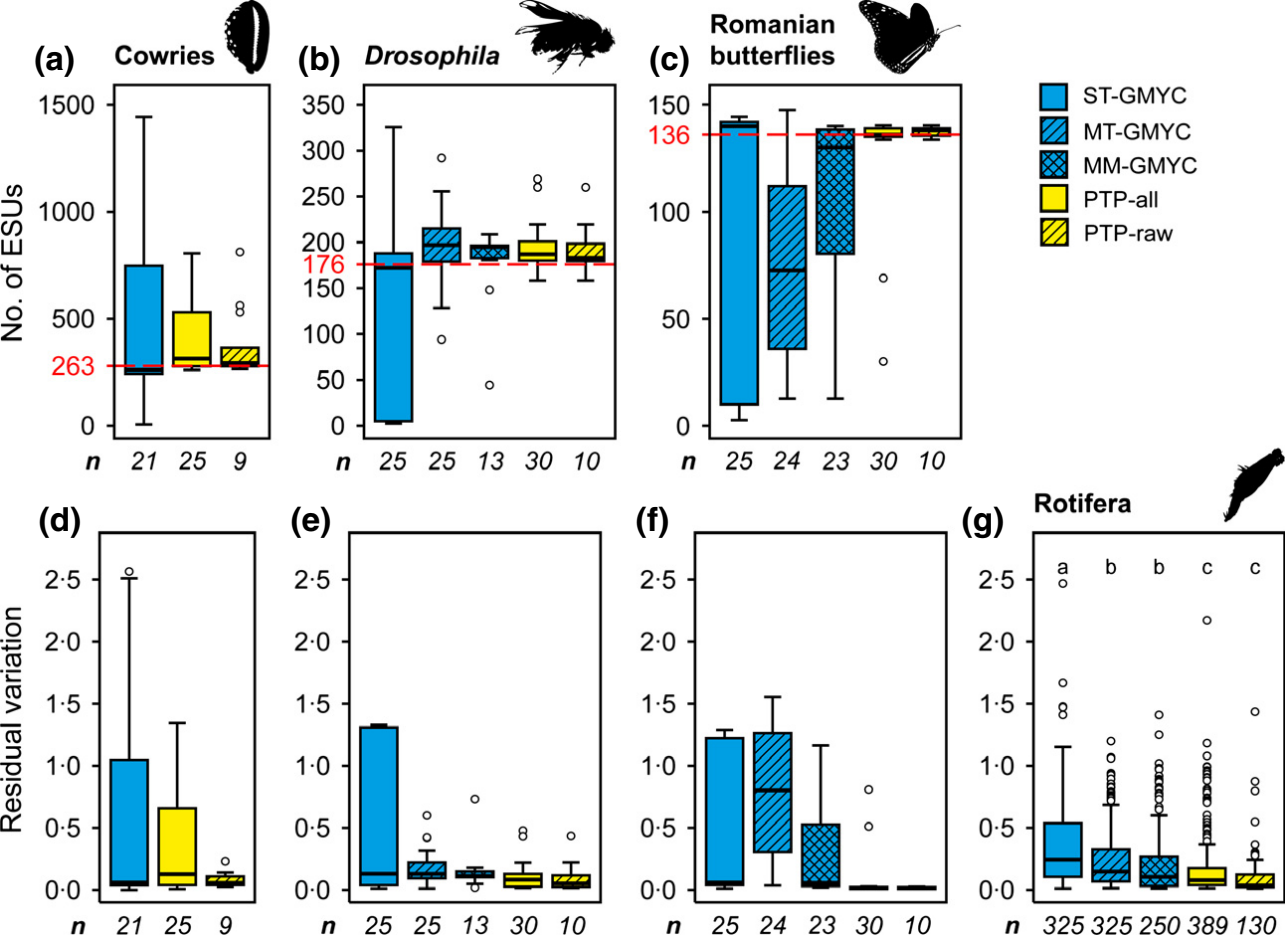
Distribution of ESU estimates (a-c) and residual variation around the expected diversity (either the traditional species count [ESUmorph; d–f] or the average ESU estimate for that clade [ESUmeanB; g]) per species delimitation method. Cowries (a,d), *Drosophila* (b,e), Romanian butterflies (c,f) and Rotifera (g), and the five species delimitation methods for each clade (ST-GMYC = single threshold, MT-GMYC = multiple thresholds, MM-GMYC = multimodel, PTP-all = all trees and PTP-raw = trees without *post hoc* smoothing) are shown separately. The traditional species count (red, dashed line), median (thick, black lines), first and third quartiles (box), 1.5 times the interquartile range (whiskers) and outliers (circles) are shown. Letters above the boxes represent significantly different comparison, and *n* below the bars represents the number of species delimitation analysis that constitutes that bar.

**Figure 2:**
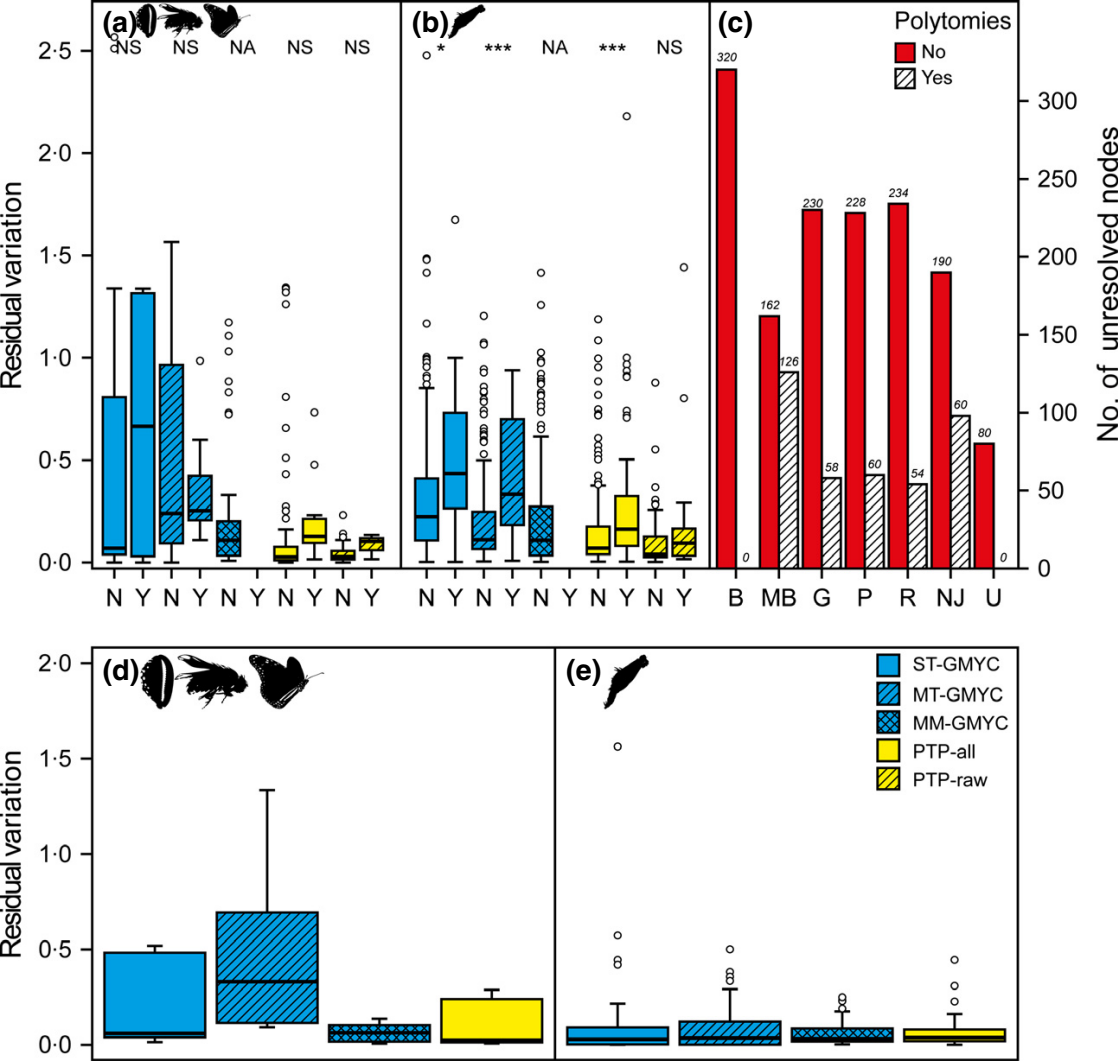
The relationship between residual variation of ESU estimates and species delimitation method when unresolved nodes are absent or present (non-Rotifera [a] and Rotifera [b]), and the number of unresolved nodes for each of the phylogenetic methods (c). The five species delimitation methods are analysed separately. Signs above the boxes denote significant differences at *P *< 0.05 (*) and *P *< 0.001 (***). NA = not applicable, NS = not significant, N = unresolved nodes are absent, Y = unresolved nodes are present, B = BEAST, MB = MrBayes, G = GARLI, P = PhyML, R = RAxML, NJ = neighbour joining and U = UPGMA.

For each data set, the number of ESUs estimated (non-Rotifera, Fig.1a–c; Rotifera, Fig. S2) and their residual variation (Table1; Fig.1d–g) varied among species delimitation methods. For all data sets, the PTP estimates, especially PTP-raw, best matched the expected diversity (Table1; Fig.1d–g). GMYC estimates varied depending on whether one or multiple thresholds or a multimodel approach was used (non-Rotifera, Fig.1a–c; Rotifera, Fig. S2); the MM-GMYC and MT-GMYC inferences were the most consistent (Fig.1d–g; Table1). The ST-GMYC estimates were more variable (non-Rotifera, Fig.1a–c; Rotifera, Fig. S2) and differed more from the expected diversity (Fig.1d–g; Table1). Reanalysis of these data without BEAST and UPGMA trees removes some of the significant differences associated with delimitation methods (Table1). For the non-Rotifera data set, there are no differences among the species delimitation methods. For the Rotifera data set, ST-GMYC is the only significantly different method (Table1). When only BEAST trees were analysed, there were no significant differences in residual variation among delimitation methods (Fig.2d–e; Table S4).

**Table 1:** Simultaneous pairwise Tukey HSD tests for general linear hypotheses. Differences in residual variation of ESU estimates between each delimitation method (Species richness) analysed separately for the non-Rotifera and Rotifera data sets, or the proportion of exact matches to the traditional species (Species identity) analysed for non-Rotifera data sets. ST-GMYC, MT-GMYC and MM-GMYC refer to GMYC using a single-, multiple-threshold and multimodel approach. PTP-all and PTP-raw refer to species delimitation analyses where either all of the trees were used, or only the trees that were not rate smoothed *post hoc*. Analyses were for (1) all of the trees and (2) the reduced data set without BEAST or UPGMA trees (for which unresolved nodes were absent)

		Species richness								Species identity			
Comparison	non-Rotifera				Rotifera				non-Rotifera			
	Estimate	SE	*Z*	*P*	Estimate	SE	*Z*	*P*	Estimate	SE	*Z*	*P*
All trees													
ST-GMYC	MT-GMYC	−0·023	0·07	−0·33	1	0·10	0·017	5·92	<0·001	0·084	0·1	0·8	0·85
ST-GMYC	MM-GMYC	0·16	0·08	2·07	0·22	0·13	0·019	6·8	<0·001	–	–	–	–
ST-GMYC	PTP	0·28	0·1	−4·80	<0·001	0·18	0·017	−10·4	<0·001	−0·18	0·081	−2·16	0·13
ST-GMYC	PTP-raw	0·25	0·09	−2·71	0·05	0·19	0·03	−7·10	<0·001	−0·12	0·12	−1·03	0·72
MT-GMYC	MM-GMYC	0·18	0·08	−2·26	0·15	0·024	0·019	−1·30	0·69	–	–	–	–
MT-GMYC	PTP	0·31	0·07	−4·54	<0·001	0·075	0·017	−4·41	<0·001	−0·26	0·1	2·55	0·05
MT-GMYC	PTP-raw	0·28	0·10	−2·77	0·042	0·09	0·03	−3·31	0·0078	−0·21	0·14	1·52	0·41
MM-GMYC	PTP	0·13	0·07	−1·70	0·42	0·051	0·018	−2·77	0·042	–	–	–	–
MM-GMYC	PTP-raw	0·097	0·10	−0·93	0·88	0·066	0·03	−2·4	0·12	–	–	–	–
PTP	PTP-raw	−0·03	0·09	0·35	1	0·014	0·02	−0·58	0·98	0·051	0·11	−0·46	0·97
Reduced data set with no BEAST or UPGMA trees													
ST-GMYC	MT-GMYC	0·16	0·25	0·63	0·97	0·18	0·038	4·74	<0·001				
ST-GMYC	MM-GMYC	−0·15	0·3	−0·43	0·99	0·17	0·04	4·22	<0·001				
ST-GMYC	PTP	0·045	0·18	−0·25	0·99	0·26	0·036	−7·162	<0·001				
ST-GMYC	PTP-raw	0·11	0·40	−0·27	0·99	0·25	0·07	−3·52	0·0034				
MT-GMYC	MM-GMYC	−0·31	0·38	0·81	0·92	−0·0033	0·041	0·08	0·99				
MT-GMYC	PTP	−0·11	0·26	0·45	0·99	0·083	0·036	−2·28	0·14				
MT-GMYC	PTP-raw	−0·052	0	0·1	1	0·07	0·07	−1·00	0·85				
MM-GMYC	PTP	0·19	0·35	−0·56	0·98	0·086	0·040	−2·14	0·19				
MM-GMYC	PTP-raw	0·26	0·50	−0·52	0·98	0·074	0·07	−1·02	0·84				
PTP-raw	PTP	0·062	0·36	−0·18	1	−0·013	0·07	0·20	0·99				
Unresolved nodes absent	Unresolved nodes present	2·95e^−5^	0·36	0	1	0·23	0·039	5·92	<0·001				

**Figure 3:**
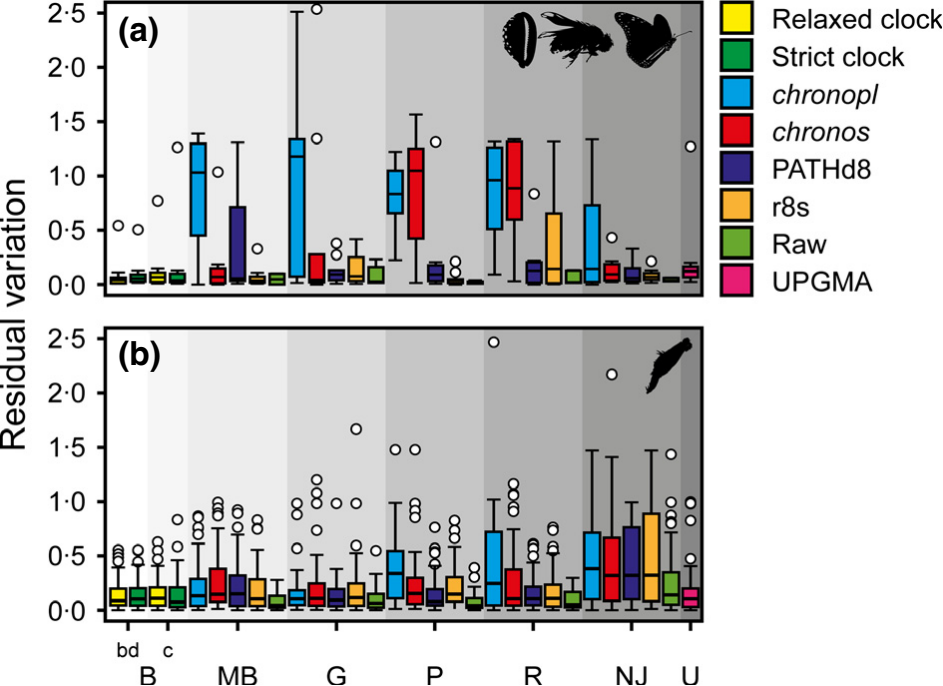
The relationship between residual variation of non-Rotifera (a) and Rotifera (b) ESU estimations pooled for all the delimitation methods with respect to the different combinations of phylogenetic and smoothing methods. Each data set was analysed using eight different phylogenetic methods (grey shaded areas). Median (thick black lines), first and third quartiles (box), 1.5 times the interquartile range (whiskers) and outliers (circles) are shown. bd = birthdeath, c = coalescent.

Different combinations of phylogenetic and smoothing methods resulted in varied ESU estimates (non-Rotifera, Fig. S3; Rotifera, Fig. S2) and residual variation (Fig.3; Fig. S4; Table S5). The tendency to under- or overestimate diversity relative to the mean varied randomly among trees smoothed with different methods (Table S6). Gene trees smoothed with the *chronopl* and *chronos* functions typically led to highly variable ESU estimates (non-Rotifera, Fig. S3; Rotifera, Fig. S2) that differed from the expected diversity (Fig.3; Fig. S4; Table S6).

### Unresolved nodes and rate heterogeneity

The proportion of unresolved nodes differed among gene trees (Fig.2c), from none for BEAST trees to 24% for NJ and 43·8% for MrBayes trees. Increased residual variation is related to the presence of unresolved nodes for Rotifera (GLMM: *t *= 5·92, df = 1046, *P *< 0·0001; Fig.2b) but not non-Rotifera (GLMM: *t *= 0, df = 81, *P *= 1; Fig.2a) clades and interacts with rate heterogeneity for both Rotifera (GLMM: *t *= −3·27, df = 1046, *P *= 0·0011) and non-Rotifera (GLMM: *t *= −3·28, df = 81, *P *= 0·0015) data sets.

### Species identity

**Figure 4:**
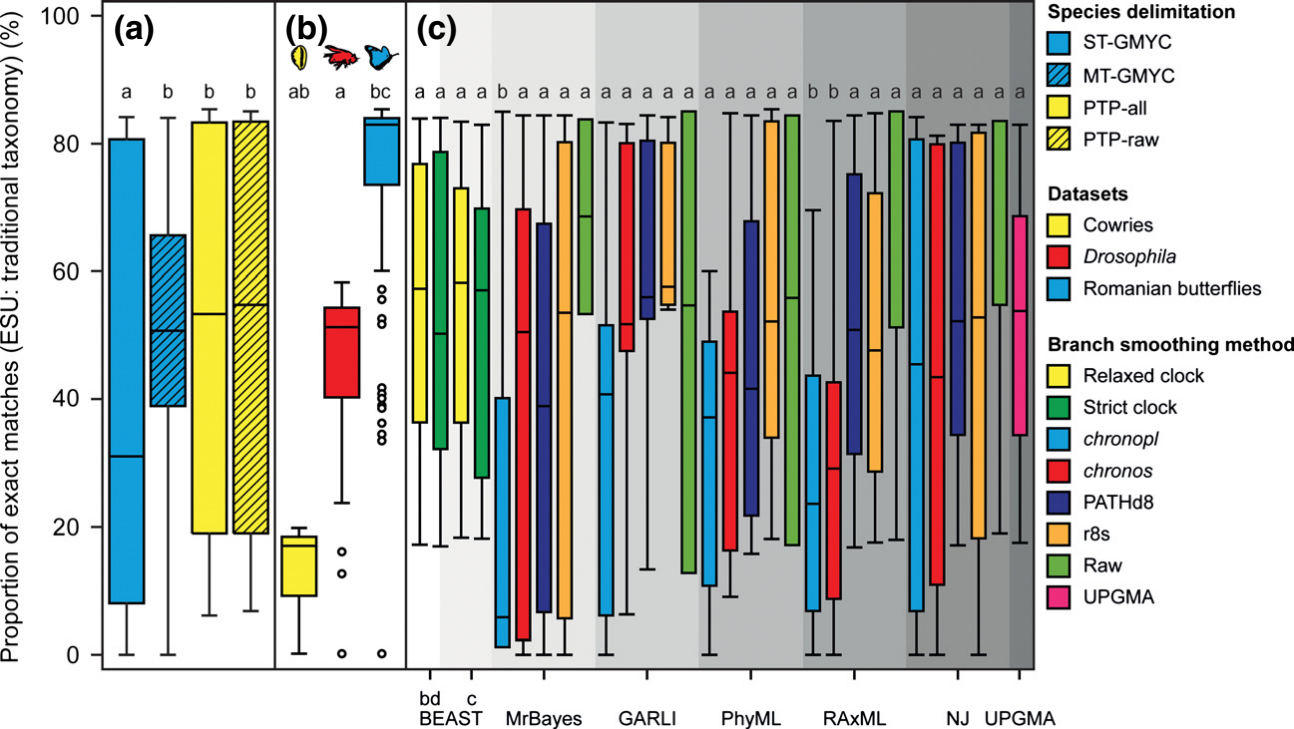
The relationship between the proportion of exact matches (morphospecies = ESU) and (a) species delimitation metric, (b) data set and (c) combination of phylogenetic and smoothing method. Each data set was analysed using eight different phylogenetic methods (grey shaded areas). Median (thick black lines), first and third quartiles (box), 1.5 times the interquartile range (whiskers) and outliers (circles) are shown. Letters above the boxes represent significantly different comparisons.

The proportion of morphospecies that were inferred as an ESU did not differ significantly among the different species delimitation methods (Fig.4; Table1). Most of the combinations of phylogenetic and smoothing methods produced similar proportions of exact matches (Table S6; Fig.4), but those smoothed with *chronopl* or *chronos* produced significantly lower proportions of exact matches, resulting from either higher levels of lumping or splitting. Differences were the largest among the data sets, with a significantly higher proportion of exact matches for the Romanian butterflies than for the cowries (GLM_binomial_: *Z* = 3·39, *P* < 0·001; Fig.4), but no differences when compared to the *Drosophila*. The proportion of exact matches was on average 63 ± 2% and was highest for the Romanian butterflies (70·4 ± 3·5%), followed by *Drosophila* (59·1 ± 2·6), and cowries (58 ± 4·4%; Fig.4; Fig. S5). The type of mismatches differed in proportion between the three data sets (Fig. S5): cowries were typically split, *Drosophila* were lumped (ST-GMYC, PTP) and split (MT-GMYC), and the Romanian butterflies were lumped.

## Discussion

Good taxonomy is central to any discipline using species as a fundamental unit. Coalescent-based, phylogenetic species delimitation clusters sequences into evolutionarily significant units. This approach relies heavily on the underlying tree and is affected by the choice of phylogenetic methods (Talavera, Dinca & Vila 2013). Our results indicate that the PTP method produces ESU estimates that are more robust to phylogenetic reconstruction methods than the GMYC method, except when BEAST trees are used.

Specifically, residual variation in ESU estimates was lowest for PTP-raw. The three implementations of the GMYC method differed in how robust they were to phylogenetic methods (MM-GMYC>MT-GMYC>ST-GMYC). As expected, the MM-GMYC produced ESU estimates that were more robust to different phylogenetic methods, although the MM-GMYC estimate is typically an average. Species delimitation using both the PTP and GMYC methods was consistent (lower residual variation) for BEAST trees, possibly because they require no *post hoc* smoothing step and contain no unresolved nodes. In contrast, analysis of NJ trees resulted in particularly large deviations in ESU estimates, irrespective of smoothing method. This is not surprising given that NJ is a clustering method that does not rely on an evolutionary model (Saitou & Nei 1987), known to underperform if the distance measure is not a correct estimate of nucleotide substitutions (Tateno, Takezaki & Nei 1994). There is also a large increase in residual variation associated with *chronopl* and *chronos* branch smoothing, which are particularly prone to haphazard lumping and splitting ESUs irrespective of the degree of between-branch smoothing (λ) chosen (File S3). This finding concurs with that of Talavera, Dinca & Vila (2013) who found that GMYC analyses of NJ trees smoothed with PATHd8, *chronopl* or *chronos* produced aberrant ESU counts.

To quantify parameters that differ among trees and may affect ESU estimates, we analysed the effect of rate heterogeneity and unresolved nodes, which are either characteristics of poor tree reconstruction (methodological or sample issues) or real features of the data. We found a significant effect of both these parameters on the GMYC and PTP output: diversity estimates for trees with highly variable rates and/or unresolved nodes deviated more widely from the expected diversity than clocklike, resolved trees (e.g. BEAST trees). Branch smoothing of trees with highly variable substitution rates can lead to exaggerated stretching of branches (Drummond & Suchard 2010), which will detriment all coalescent-based species delimitation methods that use branch lengths as an input. Unresolved nodes in the tree impinge on correct diversity estimates because their resolution can lead to artefacts in the branch length data (e.g. infinite branching rates) that could result in misplaced coalescent thresholds used for delimitation. For the GMYC, splitting might occur if infinite branching rates are found closer to the tips, while for the PTP, it might result from increased average, observed intraspecific cohesiveness resulting from no increase in branch lengths with more tips. Contrarily, the diversity could be underestimated if the unresolved nodes are closer to the root for the reciprocal reasons. Whether unresolved nodes and rate heterogeneity in the data are correlates or causes of incorrect diversity estimates remains to be tested. Encouragingly, their effect is alleviated when BEAST trees are used as input.

As a measure of how species identity differed among the methods, we assessed the proportion of ESUs that were exact matches to traditional species (morphospecies). We found similar levels of species richness to the traditional taxonomy but varying levels of discordance in identity between the traditional and DNA taxonomy. The proportion of exact matches was on average 63%, and variation in this was associated primarily, with combination of phylogenetic and smoothing methods and taxonomic group, but less with species delimitation method. We found no significant differences in the proportion of exact matches between the species delimitation methods, although the PTP method was qualitatively higher. The largest differences were between the three clades, and potentially points to the varying levels of taxonomic work in these groups. These differences seem to be driven by aberrantly deviant ESU estimates (in terms of richness and identity) associated with the use of *chronopl* and *chronos* smoothing methods, which typically split the cowrie morphospecies and lumped the *Drosophila* and Romanian butterfly species.

Traditional species of the Romanian butterflies appear to be supported by DNA taxonomy perhaps because the data set represents a geographical (rather than taxonomic) sample. Species are expected to appear more distinct in such a sample because the closest relatives of most sampled species will not be sampled (Bergsten. 2012). Although the proportion of morphospecies that were lumped, relative to split, indicates that lower intraspecific sampling in this clade is over-representing the Yule process in the tree and thus missing some of the ESUs. The higher intraspecific sampling for the cowries and *Drosophila* indicates that the splitting of these species could be associated with unresolved taxonomy (Packer. 2009) or overlapping intra- and interspecific variation (Meyer & Paulay 2005; Wiemers & Fiedler 2007). While efforts have been made to resolve the taxonomy of these groups (Meyer & Paulay 2005; O'Grady & Markow 2009), a more concerted effort is required to address the gap between DNA and traditional taxonomy across the entirety of these clades (C. Meyer pers. comm).

By assessing ESU counts across 16 data sets with over 1500 separate species delimitation analyses, we have shown that the PTP-raw model with any robust gene tree and the GMYC used on BEAST trees produce consistently robust and, on average, accurate species estimates. These findings can probably be extrapolated to other genetic markers: COI and 18S are typically used for animals (Tang. 2012, 2014), multiple markers (e.g. 16S) for bacteria (Barraclough. 2009; Morlon. 2012), ITS for fungi (Powell. 2011) and multiple markers (e.g. matK and rbcL) for plants (Hollingsworth & CBOL Plant Working Group 2009). Although the variability of these markers will likely yield different degrees of coalescent clustering and species separation (Tang. 2012) that warrants a more thorough evaluation.

Coalescent-based species delimitation is likely to gain in popularity: either to facilitate the description of biodiversity in an integrative, iterative way as a tool to tackle the burgeoning taxonomic crisis (Puillandre. 2012b), or to cluster sequences from NGS studies (Creer. 2010; Chariton. 2014). The latter would benefit from evolutionary approaches that provide a deeper understanding of the nature and extent of diversity (Barraclough. 2009). Applying coalescent-based species delimitation to NGS is currently limited by the amount of variability, the short (but ever increasing) read lengths, the amplification success of the markers used and the computational expense of the coalescent-based metrics. As with all DNA taxonomy studies, primers need to be designed to combat the low amplification success of certain primers (Zhan. 2014), robust bioinformatics pipelines need to be developed (S. Creer pers. comm.), and sampling regimes that are representative of intra- and interspecific variability and geographical range should be considered (Papadopoulou. 2008; Lohse 2009; Bergsten. 2012; Talavera, Dinca & Vila 2013).

The PTP method is appealing when speed is essential because ultrametric trees are not required (Zhang. 2013), meaning that some of the problems encountered and the additional computation required with branch smoothing may be circumvented. However, the PTP makes the assumption that branching events scale with substitutions rather than time, which might be violated when substitution rates are heterogeneous. The GMYC with a BEAST tree provided equally consistent estimates but obtaining BEAST trees is computationally expensive. However, when rate heterogeneity is high and can be adjusted across the tree estimation using models, perhaps by use of well-informed internal calibration priors, then diversity estimation might benefit from sophisticated dating and diversity estimation procedures. We feel that the GMYC is more true to the speciation process, in that speciation and coalescence happen over time and not necessarily in relation to how many substitutions occur in marker genes. While the transition between speciation and coalescent processes, used to delimit species, might be mirrored by differences in the number of species- and population-level substitutions (PTP), time is a more direct expression of the process; therefore, methods that separately model this transition (time; GMYC) and phylogenetic methods that formally correct for substitution rate variation among species (e.g. BEAST) are conceptually more appropriate. We recommend use of both PTP and GMYC methods with the appropriate phylogenetic tree or choosing between them on a case-by-case basis, bearing in mind the differences in speed and underlying theory inherent in the two methods. The PTP method with non-ultrametric trees is currently quicker to implement than the GMYC, especially the MM-GMYC, although the speed of the GMYC could be increased with parallelisation. Both the phylogenetic and species delimitation steps become computationally demanding for larger data sets (e.g. NGS studies). Such data sets, which are often taxonomically broad, are likely to violate use of a single substitution rate and so increased parameterisation and prior information is more likely to yield trees that better reflect the data and thus provide more realistic diversity estimates. We envisage that better phylogenetic handling of substitution rate heterogeneity within the samples, irrespective of delimitation method, and the use of ESU nodal support as a proxy for species identity confidence, would further improve the delimitation of primary species hypotheses from single-locus marker surveys.

## Data accessibility

All gene trees have been deposited on Dryad (Tang 2014).

## Supporting Information

**Fig. S1.** Methods overview.

**Fig. S2.** For each Rotifera clade separately, the number of ESUs delimited differs with respect to the combination of phylogenetic, smoothing, and species delimitation method (GMYC [blue] vs. PTP [yellow]).

**Fig. S3.** The relationship between the number of ESUs and different combinations of phylogenetic and smoothing method shown separately for cowries, Drosophila and Romanian butterflies. Some combinations deviate more from the morphological species count (red, dashed line) than others.

**Fig. S4.** Residual variation of ESU estimates for all 16 datasets shown separately for each species delimitation method: ST-GMYC (a), MT-GMYC (c) MM-GMYC (e), PTP-all (b) PTP-raw (d) and all together (f).

**Fig. S5.** The number of morphospecies that are exact matches (purple), lumped (orange), or split (green) relative to the ESUs.

**Table S1.** Literature review of trees used as GMYC input and the types of phylogenetic reconstruction performed from 2006 to the 11th of April, 2014).

**Table S2.** Sequences information including accession numbers, species description, publication, etc.

**Table S3.** Dataset information (# Seq., #Hap., outgroups, residual variation, etc.).

**Table S4.** Simultaneous pairwise Tukey HSD tests for General Linear Hypotheses using BEAST trees only.

**Table S5.** Simultaneous pairwise Tukey HSD tests for General Linear Hypotheses.

**Table S6.** Summary of the Linear Mixed Effects Model of the number of GMYC (single-threshold) ESUs estimated from gene trees smoothed with chronopl or chronos under varying smoothing parameters (λ).

**Data S1.**Materials and Methods. **File S1**. Does ESU_meanB_ correspond to ESU_morph_? **File S2**. Residual variation example calulation. **File S3**. Is λ a strong determinant of ESU estimation?
