# Supplementary figures and images for: Effects of phylogenetic reconstruction method on the robustness of species delimitation using single-locus data
Source: Methods Ecol Evol. 2014 Oct 29;5(10):1086–94. doi: 10.1111/2041-210X.12246 (PMC4374709; doi:10.1111/2041-210X.12246)

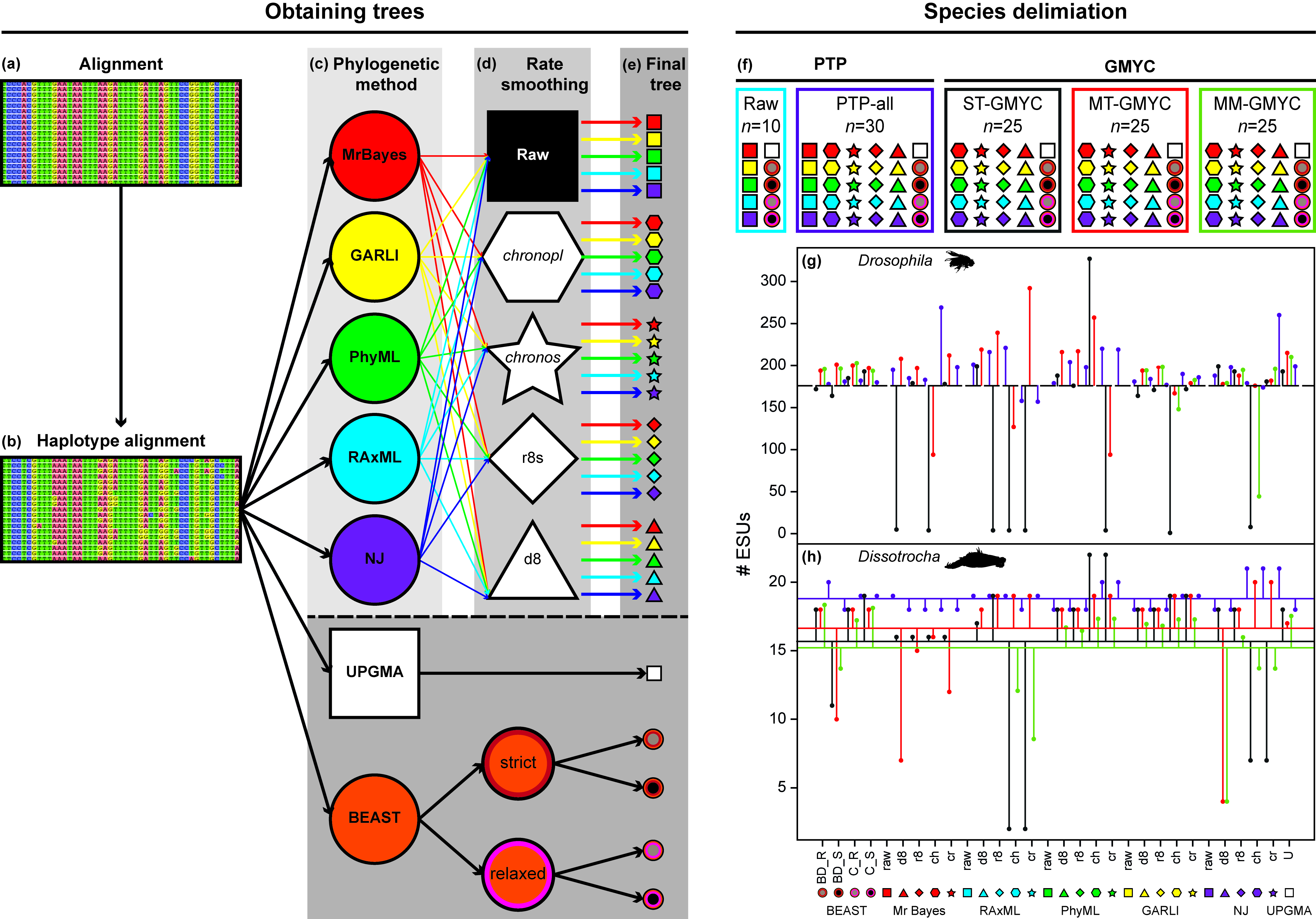

Supplement: Supplementary file 1 — Fig. S1. Methods overview. [file mee30005-1086-sd1.tif]

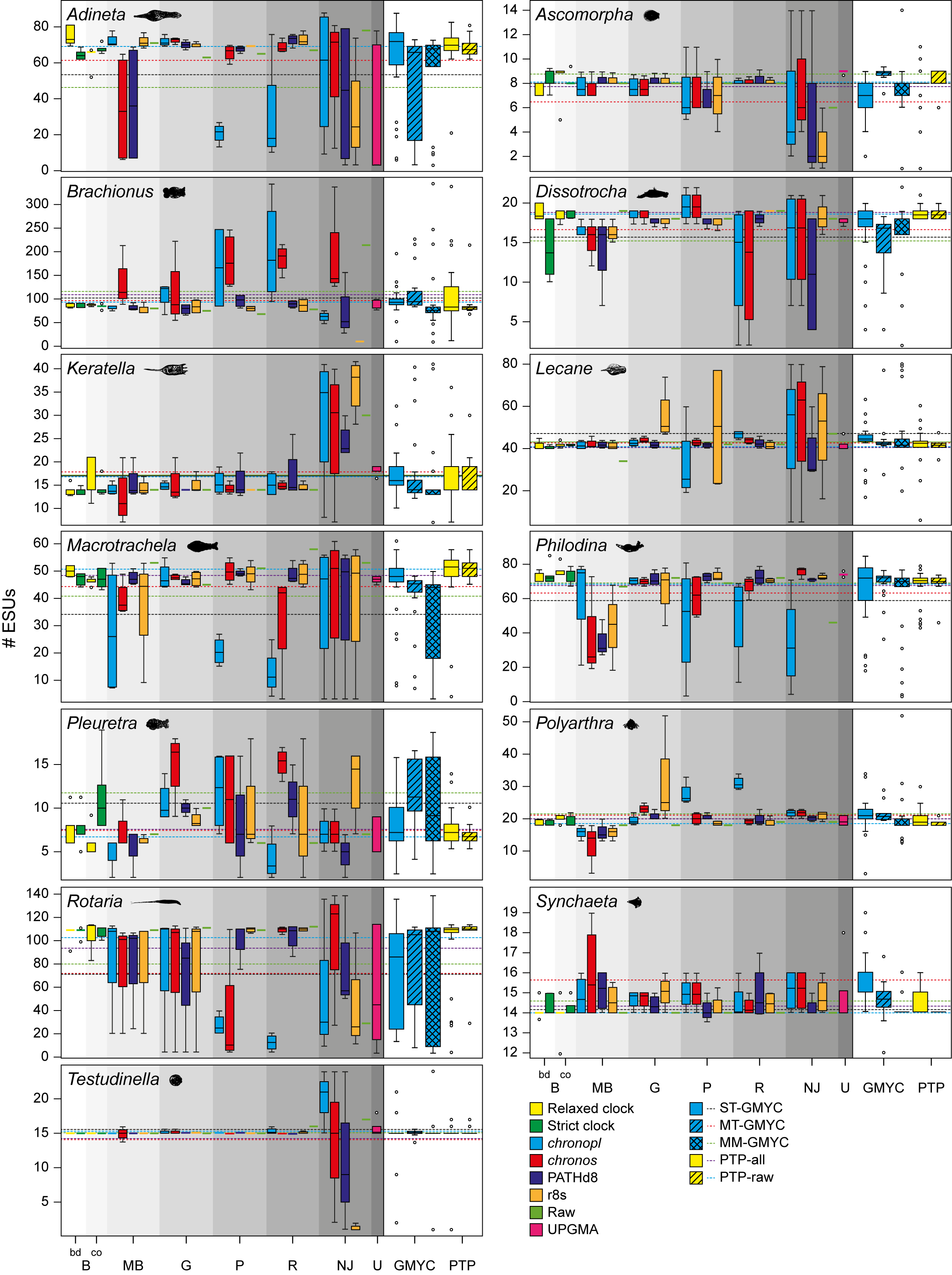

Supplement: Supplementary file 2 — Fig. S2. For each Rotifera clade separately, the number of ESUs delimited differs with respect to the combination of phylogenetic, smoothing, and species delimitation method (GMYC [blue] vs. PTP [yellow]). [file mee30005-1086-sd2.tif]

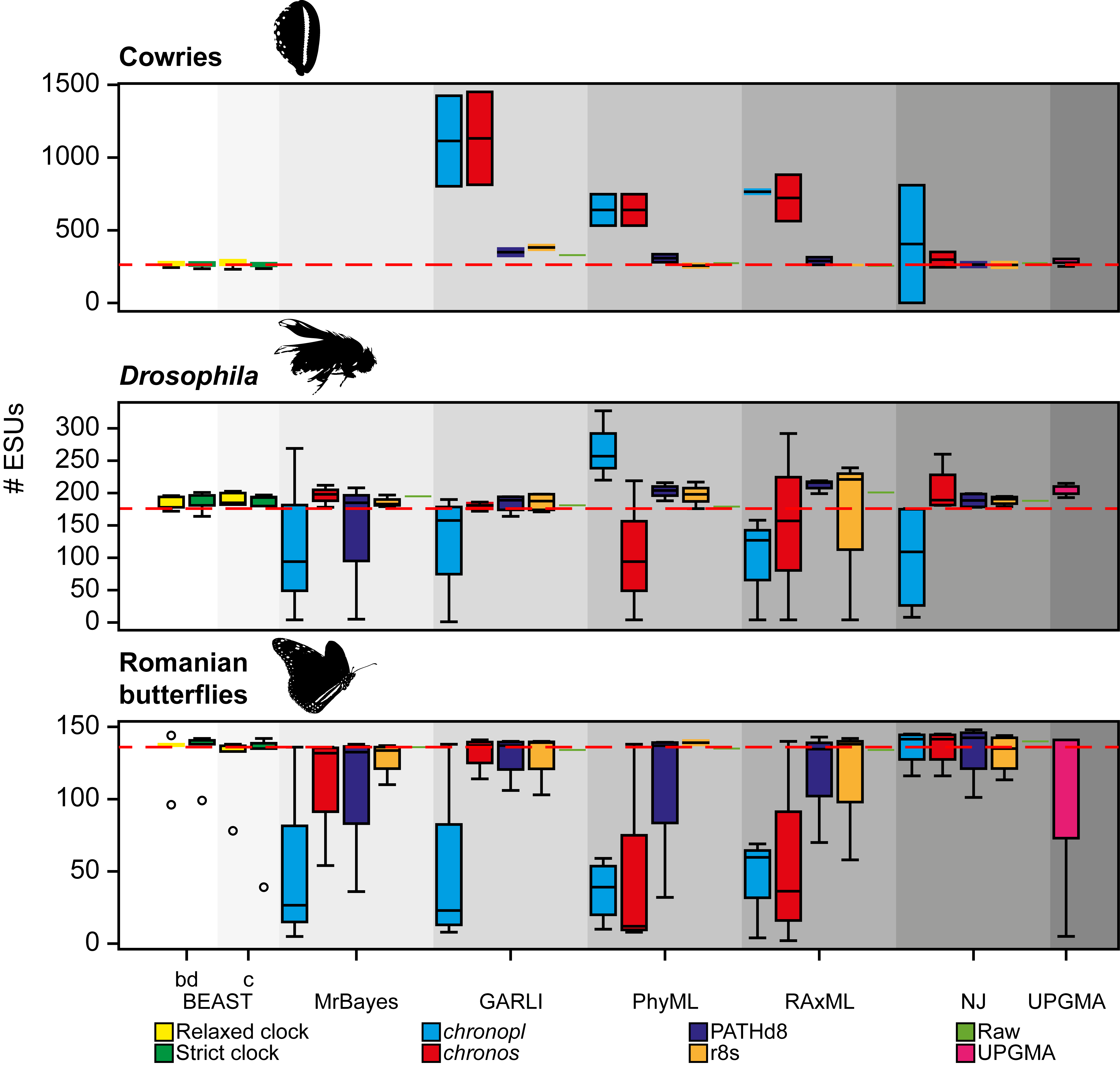

Supplement: Supplementary file 3 — Fig. S3. The relationship between the number of ESUs and different combinations of phylogenetic and smoothing method shown separately for cowries, Drosophila and Romanian butterflies. Some combinations deviate more from the morphological species count (red, dashed line) than others. [file mee30005-1086-sd3.tif]

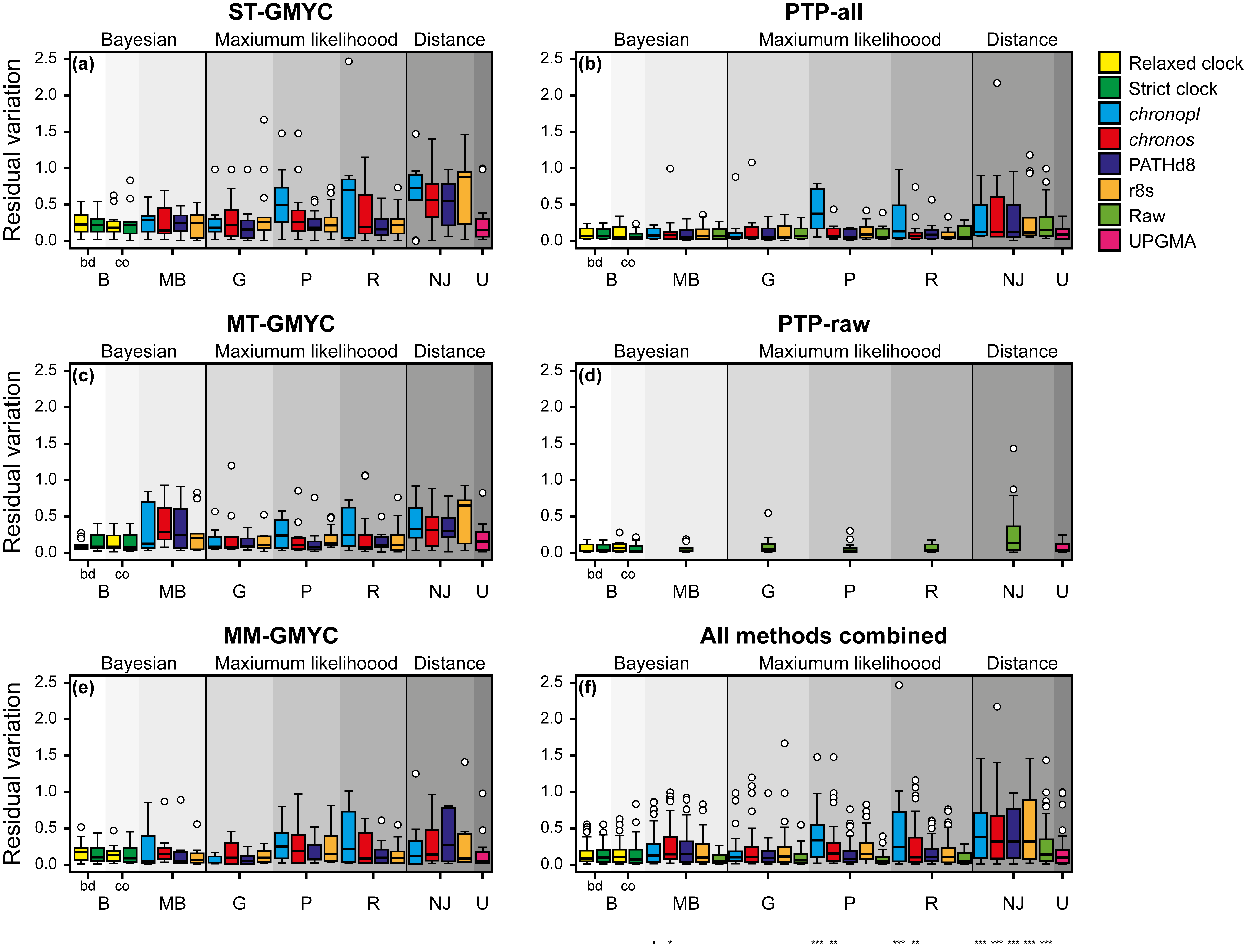

Supplement: Supplementary file 4 — Fig. S4. Residual variation of ESU estimates for all 16 datasets shown separately for each species delimitation method: ST-GMYC (a), MT-GMYC (c) MM-GMYC (e), PTP-all (b) PTP-raw (d) and all together (f). [file mee30005-1086-sd4.tif]
