# Supplementary material for: Effects of phylogenetic reconstruction method on the robustness of species delimitation using single-locus data
Source: Methods Ecol Evol. 2014 Oct 29;5(10):1086–94. doi: 10.1111/2041-210X.12246 (PMC4374709; doi:10.1111/2041-210X.12246)
Supplement: Supplementary file 12 — Data S1.Materials and Methods. File S1. Does ESUmeanB correspond to ESUmorph? File S2. Residual variation example calulation. File S3. Is λ a strong determinant of ESU estimation? [file mee30005-1086-sd12.docx]

Effects of phylogenetic reconstruction on the robustness of alternative species delimitation methods

Supplementary Information

# Supplementary Materials and Methods

Gene trees are required by both the GMYC and PTP models. Ultrametric trees are needed for the GMYC model (Pons *et al.* 2006; Fujisawa & Barraclough 2013) as it analyses branching rates, while the PTP delimits species from the number of substitutions inferred from “raw” (unsmoothed) or ultrametric trees (Zhang *et al.* 2013). Tree reconstruction followed these steps (outlined in Fig. S1): (1) align sequences, (2) remove non-unique haplotypes, (3) reconstruct gene trees, and (4) make ultrametric gene trees.

## Obtaining gene trees

### (1) Sequence alignment

Cytochrome oxidase *c* subunit 1 (COI) sequences were downloaded from GenBank (except for the cowrie dataset, which was provided by Christopher P. Meyer) and aligned with carefully selected outgroups (Table S3) using the MAFFT v6.814b plugin (Katoh *et al.* 2009) with the default settings within Geneious 5.4.6 (Drummond *et al.* 2006). Alignments were subsequently checked by eye and atypically short sequences (100bp shorter than all the others) were removed. Translation of the alignments (using the Invertebrate Mitochondrial Code) was used as an additional check of their validity. No indels, NUMTS or stop codons were found in any of the alignments. In total 10,244 sequences were obtained and aligned into 16 separate datasets representing different taxonomic ranks. The butterfly dataset was a comprehensive geographical sample (Romania) of an order (Lepidoptera; Dinca *et al.* 2011), the cowrie dataset was a comprehensive sample of a family (Cypraeidae; Meyer & Paulay 2005) and the remaining datasets were samples of genera with the exception of *Pleuretra lineata*, which is a species complex.

### (2) Removal of non-unique haplotypes

The alignments were stripped of non-unique haplotypes using DnaSP 5.10.01 (Librado & Rozas 2009), resulting in 3,190 unique haplotypes. Non-unique haplotypes were removed as the GMYC model assumes a fully bifurcating tree and zero length terminal branches hamper the likelihood estimation (Fujisawa & Barraclough 2013). Collapsing datasets is typically the first step in tree based species delimitation analyses and does not significantly affect the outcome of the GMYC analysis (Talavera *et al.* 2013). Performing this step before both GMYC and PTP analyses allowed for direct comparisons between the methods at reduced computation expense.

### (3) Reconstruction of gene trees

Haplotype alignments were used to generate gene trees using distance, maximum likelihood (ML) and Bayesian inference (BI) methods. For each haplotype alignment, 10 trees were reconstructed using different methods with the most commonly used software. Distance trees were reconstructed using Neighbour Joining (NJ; Saitou & Nei 1987) and UPGMA (Un-weighted Pair Group Method with Arithmetic mean; Sokal & Michener 1958). NJ trees were generated using the *ape* 3.0.7 package (Paradis *et al.* 2004) in R 2.15.2 (R Core Team 2012) from sequence distance matrices computed by the Kimura (K80 or K2P) model (Kimura 1980). UPGMA distance trees were built with MEGA5 (Tamura *et al.* 2011) using p-distances instead of a model of evolution. For the ML and BI methods, gene trees were reconstructed using the best model of evolution as determined *a priori* using the lowest Akaike Information Criterion (AIC computed in jModelTest 2 - Darriba *et al.* 2012; Table S3). ML gene trees were reconstructed using GARLI 2.01 (Zwickl 2006), PhyML 3.0 (Guindon *et al.* 2010) and RAxML (Stamatakis 2006). GARLI trees were reconstructed with the evolutionary models with the lowest AIC score and 5,000,000 generations. All PhyML trees were generated using the ATGC online server. For each analysis, the appropriate evolutionary model was selected with optimised equilibrium frequencies and optimal trees were searched for using the combined nearest neighbour and subtree pruning and regrafting tree rearrangement option (available in PhyML 3.0), but otherwise default settings were used. RAxML trees were generated using either the RAxML BlackBox webserver (Stamatakis 2006; Stamatakis *et al.* 2008) or using the CIPRES Science Gateway server (Miller *et al.* 2010) with a Gamma model of rate heterogeneity, maximum likelihood model search and an estimated proportion of invariable sites.

BI trees were reconstructed using MrBayes 3.2.1 (Huelsenbeck & Ronquist 2001; Ronquist *et al.* 2012) and BEAST v1.7.5 (Drummond & Rambaut 2007). MrBayes analyses were run for 10,000,000 generations with four parallel searches sampling every 500 generations. Consensus trees were generated using the *sumt* command with a 10% burnin, but only if the average standard deviation of the chains was less than 0.01 (as recommended in the MrBayes documentation). Four separate BEAST runs, with different parameters, were performed for each clade. BEAST input files were generated using BEAUti v1.7.5, each search ran with a substitution rate of 1.76% per million years, the most accepted rate for most invertebrates (Wilke *et al.* 2009) under either an uncorrelated lognormal relaxed molecular clock or a strict molecular clock and either a ‘Speciation: Birth-Death Process’ (Gernhard 2008) or a ‘Coalescent’ tree prior. Each dataset was run for at least 30,000,000 generations sampling every 1,000 steps on the CIPRES Science Gateway server. All of the other options were kept as the BEAUti default settings. The effective sample size (ESS) of each run was determined using Tracer v1.5 (Rambaut & Drummond 2007) and only trees with an ESS of at least 200 were kept (as recommended in the BEAST manual). Initially low ESS scores for some runs were resolved by increasing the number of generations of MCMC sampling. The burnin was set to 10% and TreeAnnotator v1.6.1 was used to summarise the trees to give a maximum clade credibility tree with target node heights.

### (4) Make ultrametric gene trees

Ultrametric trees are the required input for the GMYC model as branch lengths need to convey time; although not required by the PTP, ultrametric trees were also analysed using the PTP for a direct comparison. Of the ten gene trees produced for each clade, five were ultrametric by default (the four BEAST combinations and UPGMA), while the remaining five (MrBayes, GARLI, PhyML, RAxML and NJ) were smoothed using four different methods (r8s - Sanderson 2003; the *ape* functions: *chronopl* and *chronos* - Paradis *et al.* 2004; and PATHd8 - Britton *et al.* 2007). These four branch smoothing methods were used independently on each raw gene tree to generate ultrametric input trees; this totalled 20 trees per clade. The r8s software was used to perform nonparametric branch smoothing (NPRS). This method smoothes rate changes among lineages while penalising fast rate changes from mother to daughter. The level of smoothing was optimised using a cross validation procedure. PATHd8 uses mean path length to smooth substitution rates between sister groups by sequentially averaging path lengths from an internode to all the descending terminals. The R package *ape* has two branch smoothing functions: *chronopl* (semi-parametric penalised likelihood) and *chronos* (an update of *chronopl*). For each of these functions a smoothing parameter, lambda (λ), needs to be set. Lambda controls the trade-off between branches having their own rate (λ → 0) and minimising rate changes between connecting branches (λ → 1). Rates vary more among branches as λ tends towards zero, while the variation among branches is more clock-like as λ tends towards one. For all analyses, λ was set to 1 as varying λ did not consistently affect the numbers of ESUs obtained (Supplementary File S2).

## Species delimitation

Outgroup taxa were pruned from each tree before species delimitation analysis. For each dataset, the ten raw gene trees produced in (3) and the 20 additional smoothed trees produced in (4) were analysed with the PTP method (*n* = 30). The five ultrametric trees (BEAST and UPGMA) produced in (3) and the 20 additional smoothed trees produced in (4) were analysed with the GMYC method (*n* = 25). The GMYC and PTP methods delimit species defined as independently evolving, monophyletic units (an operational version of the Evolutionary Species Concept, Simpson 1951). Both methods model species- and population-level processes, separately, and identify a transition point between the two; this threshold is subsequently used to delimit ESUs.

### Generalized Mixed Yule Coalescent model

The GMYC takes advantage of the pattern expected if species have been evolving independently for a sufficiently long period of time, namely distinct genetic clusters separated in genospace by long internal branches (Barraclough & Nee 2001). An ultrametric gene tree (where branch lengths are equivalent or relative to time) well-sampled for both intra- and interspecific variation will bifurcate in a predictable manner: species-level processes will lead to slower branching rates while population-level coalescent processes will exhibit accelerated branching patterns. It follows that there should be a transition in branching rate on a tree between species level processes (e.g. speciation and extinction) and population level processes (coalescence of alleles). The GMYC is a likelihood-based method that models the branching rate of both diversification between species (Yule model - Yule 1925; Nee *et al.* 1994) and genealogical branching within populations (neutral coalescent - Hudson 1990) to identify from the data whether there is a significant shift (threshold) in branching rate. A *Χ*^2^ test is performed to gauge the significance of the application of the GMYC against the null hypothesis of a single coalescent with one branching rate. If significant, the threshold is used to delimit species on the gene tree. The GMYC method allows for either a single or multiple thresholds at which branching rates shift. Multiple-threshold allows for depth of the transition from coalescent to speciation to vary among lineages (Monaghan *et al.* 2009; Fujisawa & Barraclough 2013), and so should better accommodate rate heterogeneity within datasets. The multiple-threshold algorithm assesses the likelihood of new clusters by either splitting or lumping existing clusters and identifying increases in likelihood improvement. Clusters that increase the likelihood of the model are chosen as new starting points for the next heuristic search.

Both single- and multiple-threshold GMYC methods search for the model with the highest likelihood score, however, the most likely solution may not be the only model with which the null model (single coalescent) can be rejected. Powell (2012) and Fujisawa and Barraclough (2013) implemented an alternative GMYC approach that assigns weights to and ranks all alternative models that reject the null model based on their fit to the data using AIC (Powell’s GMYC multimodel script is available from http://dx.doi.org/10.1111/j.2024-210X.2011.00122.x). The Powell multimodel GMYC approach uses model averaging to infer parameters and species probabilities. Each ultrametric tree was analysed by the GMYC with three different approaches: single threshold (ST-GMYC), multiple thresholds (MT-GMYC), and multimodel (MM-GMYC). These analyses were performed with the *splits* 1.0–11 package (Ezard *et al.* 2009 - available from https://r-forge.r-project.org/ projects/splits) in R.

### Poisson Tree Process (PTP)

The PTP method does not rely on ultrametric trees as input. Instead, the PTP model delimits species directly from the number of substitutions inferred by the branch lengths, which represent the mean expected number of substitutions per site between two branching events. The fundamental assumption is that the intraspecific number of substitutions will be significantly lower than the interspecific number of substitutions. It is assumed that a gene tree sampled both intra- and interspecifically will have been generated by two independent Poisson tree processes (species- and population-level). Support for only one PTP process could mean that only a single population exists or that each tip is a separate species. Note that this implicitly assumes that molecular substitution rates are constant across the tree: if they varied considerably then variation in substitution rates across the tree could impair correct identification of the transition from species- to population-level processes. A standard likelihood-ratio test with one degree of freedom is performed to test for multiple classes of branch length and, if present, a maximum likelihood search returns the most likely speciation rate, coalescent rate and the transition between them. The PTP model was used for each of the rooted trees (raw and ultrametric), using the PTP web server (http://species.h-its.org/ptp/) and a mimimum *P*-value of 0.05. For the ultrametric trees, the PTP should still use the branch lengths to represent number of substitutions between branching events but if the branch lengths are not a good representation of the underlying substitution rates, then the PTP will be biased by that assumption.

# Supplementary File S1

## Does ESU_meanB_ correspond to ESU_morph_?

Residual variation is calculated as:

|ESU*_X_***-**ESU_expected_| ÷ ESU_meanA_

where ESU_expected_ is either the morphological species count (ESU_morph_) or the mean ESU estimate of the phylogenetic methods (ESU_meanB_). The use of ESU_meanB_ as a proxy for ESU_morph_ was validated using the three datasets where residual variation could be calculated using both ESU_morph_ and ESU_meanB_ (cowries, *Drosophila* and Romanian butterflies). Residual variation using either ESU_morph_ and ESU_meanB_ never deviated by more than 8.5% and differences were on average 1.54± 0.78% within each other (Fig. S6).

# Supplementary File S2

## Residual variation example calulation

Residual variation is calculated as:

|ESU*_X_***-**ESU_expected_| ÷ ESU_meanA_

For the cowries, *Drosophila*, and butterflies, the morphological species count (ESU_morph_; as determined by GenBank species names) was used as the measure for expected diversity. As an example: the number of *Drosophila* ESUs identified using the PTP method using the RAxML tree smoothed using r8s (i.e. 221) was subtracted from the morphospecies count (i.e. 176) and divided by the average *Drosophila* ESU count for all of the PTP estimates (|221-176|÷194.66=0.23). Therefore, for this particular delimitation method and combination of phylogenetic reconstruction and branch smoothing method, the residual variation is 0.23.

For the Rotifera datasets, the mean species estimate from all of the methods excluding the focal estimate (ESU_meanB_) was used as the measure of expected diversity. For example: for *Dissotrocha* (Bdelloidea: Rotifera), the number of ESUs identified using the PTP method on the RAxML tree smoothed using r8s (i.e. 19) was subtracted from the average PTP ESU estimate across the other 29 estimates (i.e. 18.79) and divided by the average *Dissotrocha* ESU count for all of the PTP estimates (|19-18.79|÷18.8=0.011). For this particular delimitation method and combination of phylogenetic reconstruction and branch smoothing method, the residual variation is 0.11.

This was repeated for each combination of species delimitation, phylogenetic reconstruction and branch smoothing method (Fig. S1g,h) and then for each dataset.

# Supplementary File S3

## Is λ a strong determinant of ESU estimation?

To ascertain the effect of changing the smoothing parameter, λ, on the ESU estimation of *chronopl* and *chronos* trees, we generated ten *chronopl* and ten *chronos* ultrametric trees per raw tree with λ values evenly distributed from 0.1 to 1.0 and estimated the diversity in each. This resulted in 1,560 ultrametric trees (minus the cowrie MrBayes and GARLI trees which did not converge and failed, respectively). For each one of these ultrametric trees, we delimited the number of ESUs using the ST-GMYC. We analysed the effect of λ on the estimated diversity using Generalized Linear Mixed Models via Penalised Quasi-Likelihood (Venables & Ripley 2002) with a quasiPoisson error structure to account for the overdispersed count data (ESU*_X_*). The number of ESUs was used as the response variable and the branch smoothing method (*chronopl* or *chronos*), λ value and phylogenetic reconstruction method (five levels) as explanatory variables, while blocking out clades as a random effect. We found that there is substantial variation in ESU estimates for some datasets but not a consistent effect of λ on this variation. Phylogenetic reconstruction and branch smoothing method are much stronger determinants of ESU estimates (Table S6; Fig. S5).

# References

Barraclough, T.G. & Nee, S. (2001). Phylogenetics and speciation. *Trends in Ecology & Evolution*, **16**, 391–399.

Britton, T., Anderson, C.L., Jacquet, D., Lundqvist, S. & Bremer, K. (2007). Estimating divergence times in large phylogenetic trees. *Systematic Biology*, **56**, 741–52.

Darriba, D., Taboada, G.L., Doallo, R. & Posada, D. (2012). jModelTest 2: more models, new heuristics and parallel computing. *Nature Methods*, **9**, 772.

Dinca, V., Zakharov, E. V, Hebert, P.D.N. & Vila, R. (2011). Complete DNA barcode reference library for a country’s butterfly fauna reveals high performance for temperate Europe. *Proceedings of the Royal Society London B*, **278**, 347–55.

Drummond, A.J., Kearse, M., Heled, J., Moir, R., Thierer, T., Ashton, B., Wilson, A. & Stones-Havas, S. (2006). Geneious v5.4.2. Available from http://www.geneious.com.

Drummond, A.J. & Rambaut, A. (2007). BEAST: Bayesian evolutionary analysis by sampling trees. *BMC Evolutionary Biology*, **7**, 214.

Ezard, T.H.G., Fujisawa, T. & Barraclough, T.G. (2009). splits: SPecies’ LImits by Threshold Statistics. http://R–Forge.R–project.org/projects/splits/.

Fujisawa, T. & Barraclough, T.G. (2013). Delimiting species using single-locus data and the Generalized Mixed Yule Coalescent (GMYC) approach: A revised method and evaluation on simulated datasets. *Systematic Biology*, **62**, 707–724.

Gernhard, T. (2008). The conditioned reconstructed process. *Journal of Theoretical Biology*, **253**, 769–78.

Guindon, S., Dufayard, J.-F., Lefort, V., Anisimova, M., Hordijk, W. & Gascuel, O. (2010). New algorithms and methods to estimate maximum-likelihood phylogenies: assessing the performance of PhyML 3.0. *Systematic Biology*, **59**, 307–21.

Hudson, R.R. (1990). Gene genealogies and the coalescent process. *Oxford Surveys in Evolutionary Biology*, **7**, 1–44.

Huelsenbeck, J.P. & Ronquist, F. (2001). MRBAYES: Bayesian inference of phylogenetic trees. *Bioinformatics*, **17**, 754–7.

Katoh, K., Asimenos, G. & Toh, H. (2009). Multiple alignment of DNA sequences with MAFFT. *Methods in Molecular Biology*, **537**, 39–64.

Kimura, M. (1980). A simple method for estimating evolutionary rates of base substitutions through comparative studies of nucleotide sequences. *Journal of Molecular Evolution*, **16**, 111–120.

Librado, P. & Rozas, J. (2009). DnaSP v5: A software for comprehensive analysis of DNA polymorphism data. *Bioinformatics*, **25**, 1451–1452.

Meyer, C.P. & Paulay, G. (2005). DNA barcoding: error rates based on comprehensive sampling. *PLoS Biology*, **3**, e422.

Miller, M.A., Pfeiffer, W. & Schwartz, T. (2010). Creating the CIPRES Science Gateway for inference of large phylogenetic trees. *2010 Gateway Computing Environments Workshop* pp. 45–52. Institute of Electrical and Electronics Engineers (IEEE), New Orleans, Louisiana, USA.

Monaghan, M.T., Wild, R., Elliot, M., Fujisawa, T., Balke, M., Inward, D.J.G., Lees, D.C., Ranaivosolo, R., Eggleton, P., Barraclough, T.G. & Vogler, A.P. (2009). Accelerated species inventory on Madagascar using coalescent-based models of species delineation. *Systematic Biology*, **58**, 298–311.

Nee, S., May, R.M. & Harvey, P.H. (1994). The reconstructed evolutionary process. *Philosophical Transactions of the Royal Society of London. Series B, Biological sciences*, **344**, 305–11.

Paradis, E., Claude, J. & Strimmer, K. (2004). APE: Analyses of Phylogenetics and Evolution in R language. *Bioinformatics*, **20**, 289–290.

Pons, J., Barraclough, T.G., Gomez-Zurita, J., Cardoso, A., Duran, D., Hazell, S., Kamoun, S., Sumlin, W. & Vogler, A.P. (2006). Sequence-based species delimitation for the DNA taxonomy of undescribed insects. *Systematic Biology*, **55**, 595–609.

Powell, J.R. (2012). Accounting for uncertainty in species delineation during the analysis of environmental DNA sequence data. *Methods in Ecology and Evolution*, **3**, 1–11.

R Core Team. (2012). *R: A language and environment for statistical computing*. R Foundation for Statistical Computing, Vienna, Austria.

Rambaut, A. & Drummond, A.J. (2007). Tracer v1.4. Retrieved from http://beast.bio.ed.ac.uk/Tracer

Ronquist, F., Teslenko, M., van der Mark, P., Ayres, D.L., Darling, A., Höhna, S., Larget, B., Liu, L., Suchard, M. a & Huelsenbeck, J.P. (2012). MrBayes 3.2: efficient Bayesian phylogenetic inference and model choice across a large model space. *Systematic Biology*, **61**, 539–42.

Saitou, N. & Nei, M. (1987). The neighbor-joining method: a new method for reconstructing phylogenetic trees. *Molecular Biology and Evolution*, **4**, 406–25.

Sanderson, M.J. (2003). r8s: Inferring absolute rates of molecular evolution and divergence times in the absence of a molecular clock. *Bioinformatics*, **19**, 301–302.

Simpson, G.G. (1951). The species concept. *Evolution*, **5**, 285–298.

Sokal, R. & Michener, C. (1958). A statistical method for evaluating systematic relationships. *University of Kansas Science Bulletin*, **38**, 1409–1438.

Stamatakis, A. (2006). RAxML-VI-HPC: maximum likelihood-based phylogenetic analyses with thousands of taxa and mixed models. *Bioinformatics*, **22**, 2688–90.

Stamatakis, A., Hoover, P. & Rougemont, J. (2008). A rapid bootstrap algorithm for the RAxML Web servers. *Systematic Biology*, **57**, 758–71.

Talavera, G., Dinca, V. & Vila, R. (2013). Factors affecting species delimitations with the GMYC model: insights from a butterfly survey. *Methods in Ecology and Evolution*, **4**, 1101–1110.

Tamura, K., Peterson, D., Peterson, N., Stecher, G., Nei, M. & Kumar, S. (2011). MEGA5: molecular evolutionary genetics analysis using maximum likelihood, evolutionary distance, and maximum parsimony methods. *Molecular Biology and Evolution*, **28**, 2731–9.

Venables, W.N. & Ripley, B.D. (2002). *Modern Applied Statistics with S.*, 4th edn. Springer, New York.

Wilke, T., Schultheiß, R. & Albrecht, C. (2009). As time goes by: A simple fool’s guide to molecular clock approaches in invertebrates. *American Malacological Bulletin*, **27**, 25–45.

Yule, G.U. (1925). A mathematical theory of evolution, based on the conclusions of Dr. JC Willis, FRS. *Philosophical Transactions of the Royal Society of London. Series B, Biological sciences*, **213**, 21–87.

Zhang, J., Kapli, P., Pavlidis, P. & Stamatakis, A. (2013). A general species delimitation method with applications to phylogenetic placements. *Bioinformatics*, **29**, 2869–76.

Zwickl, D.J. (2006). *GARLI: genetic algorithm for rapid likelihood inference*. University Texas at Austin.
