# Supplementary material for: Effects of phylogenetic reconstruction method on the robustness of species delimitation using single-locus data
Source: Methods Ecol Evol. 2014 Oct 29;5(10):1086–94. doi: 10.1111/2041-210X.12246 (PMC4374709; doi:10.1111/2041-210X.12246)
Supplement: Supplementary file 13 [file mee30005-1086-sd13.docx]

Figure S1: Methods overview. For each of the 16 datasets, an alignment with suitable outgroups is made (a) and collapsed into an alignment of unique haplotypes (b). This haplotype alignment is analysed with ten different phylogenetic methods (c). Of these, five produce ultrametric trees (UPGMA and BEAST), while the other five are either left 'raw' or require smoothing with one of four smoothing methods (d). In total, 30 trees (e) are generated. These final trees are analysed using the PTP and GMYC species delimitation methods (f). Note that non-ultrametric trees can be analysed by the PTP but not the GMYC method. Species delimitation analyses are split into PTP using unsmoothed trees only (as intended by the authors [PTP-raw]), PTP with all trees [PTP-all], GMYC with a single (ST-GMYC) or multiple (MT-GMYC) thresholds and a multimodel approach (MM-GMYC), applied to n trees in total. The robustness of each delimitation method (using *Drosophila* [g] and Dissotrocha [a rotifer; h] as examples): the absolute distance of the ESU estimate (ESU*_X_*) to the expected ESU count (either morphological species number, ESU_morph_, [g] or the average ESU estimate for that particular delimitation method, ESU_meanB_, [h]).

Figure S2: For each Rotifera clade separately, the number of ESUs delimited differs with respect to the combination of phylogenetic, smoothing, and species delimitation method (GMYC [blue] vs. PTP [yellow]). Some combinations deviate more from the expected diversity (ESU_meanB_) than others. The average for each species delimitation method (dotted lines) is shown: ST-GMYC (black), MT-GMYC (red), MM-GMYC (green), PTP-all (purple), and PTP-raw (blue). The grey shaded areas correspond to the different phylogenetic methods. Median (thick black lines), first and third quartiles (box), 1.5 times the interquartile range (whiskers), and outliers (circles) are shown. For results with no variation, a single line of the corresponding colour is used instead of a box. Abbreviations: bd = birthdeath, c = coalescent, B = BEAST, MB = MrBayes, G = GARLI, P = PhyML, R = RAxML, NJ = neighbour joining and U = UPGMA.

Figure S3: The relationship between the number of ESUs and different combinations of phylogenetic and smoothing method shown separately for cowries, *Drosophila* and Romanian butterflies. Some combinations deviate more from the morphological species count (red, dashed line) than others. No data are available for the cowries dataset reconstructed with MrBayes. The grey shaded areas correspond to the different phylogenetic methods. Median (thick black lines), first and third quartiles (box), 1.5 times the interquartile range (whiskers), and outliers (circles) are shown. Abbreviations: bd = birthdeath, c = coalescent, NJ = neighbour joining.

Figure S4: Residual variation of ESU estimates for all 16 datasets shown separately for each species delimitation method: ST-GMYC (a), MT-GMYC (c) MM-GMYC (e), PTP-all (b) PTP-raw (d) and all together (f). Each dataset was analysed by Bayesian, maximum likelihood and distance methods using eight different phylogenetic methods (BEAST [B] with either a birthdeath [bd] or coalescent [co] tree prior, MrBayes [MB], GARLI [G], PhyML [P], RAxML [R], neighbour joining [NJ] and UPGMA [U]). The grey shaded areas correspond to the different phylogenetic methods. Median (thick, black lines), first and third quartiles (box), 1.5 times the interquartile range (whiskers), and outliers (circles) are shown.

Figure S5: The number of morphospecies that are exact matches (purple), lumped (orange), or split (green) relative to the ESUs. Species delimitation methods are shown separately (ST-GMYC with a single threshold, MT-GMYC with multiple thresholds, and PTP [PTP-raw are denoted with a † above the bar]). The MM-GMYC was not analysed as it averages ESU counts over multiple models and so does not return ESU counts as integers. Cowries (a; 263 morphospecies species), *Drosophila* spp. (b; 176 morphospecies species), and Romanian butterflies (c; 136 morphospecies species) are shown separately. Each dataset was analysed using eight different phylogenetic methods (grey shaded areas) and nine different rate smoothing methods; the specific combination of phylogenetic and rate smoothing methods is shown below each bar. Abbreviations: bdr = birthdeath with a relaxed molecular clock, bds = birthdeath with a strict molecular clock, cr = coalescent with a relaxed molecular clock, cs = coalescent with a strict molecular clock, *cpl* = *chronopl*, *cos* = *chronos*, d8 = PATHd8, r8s = r8s, B = BEAST, MB = MrBayes, G = GARLI, P = PhyML, R = RAxML, NJ = neighbour joining, U = UPGMA. Where species delimitation was not possible, no data are shown (*).
